# Pancreatic intraductal papillary mucinous neoplasm with invasive carcinoma and uterine metastasis: a case report

**DOI:** 10.3389/fonc.2025.1562588

**Published:** 2026-02-24

**Authors:** Yingxue Guo, Yuan Wang, Fanghua Li, Yanxia Liu, Yixiu Han, Yingyun Zhang, Xiaolong Miao, Feifei Zhao

**Affiliations:** 1 Department of Oncology, Shengli Oilfield Central Hospital, Dongying, China; 2 Department of Surgery, Shengli Oilfield Central Hospital, Dongying, China; 3 Department of Surgery, The Affiliated Hospital Of QingDao University, Qingdao, China

**Keywords:** chemotherapy, debulking surgery, intraductal papillary mucinous neoplasm, pancreatic cancer, uterine metastasis

## Abstract

Intraductal papillary mucinous neoplasm (IPMN) with invasive carcinoma is a rare type of pancreatic cancer that has a better prognosis than classic pancreatic infiltrating ductal carcinoma. Most distant metastases occur in the lymph nodes, lungs, liver, and bones at advanced stages. We report a rare case of an IPMN with invasive carcinoma that metastasized to the uterus, resulting in long-term survival after debulking surgery combined with hyperthermic intraperitoneal chemotherapy on a systemic treatment basis. This rare case highlights the need for oncologists and gynecologists to be vigilant regarding these uncommon metastatic diseases and exercise caution in diagnosis. Comprehensive treatments, including debulking surgery, may improve survival.

## Introduction

Intraductal papillary mucinous neoplasm (IPMN) with invasive carcinoma is a rare type of pancreatic cancer and has a better prognosis compared to classic infiltrating ductal carcinoma of the pancreas. Most distant metastases occur in lymph nodes, lung, liver, and bone at advanced stages. We report a rare case of an IPMN with invasive carcinoma that metastasized to the uterus, achieving long-term survival after debulking surgery combined with hyperthermic intraperitoneal chemotherapy as part of a systemic treatment approach.

## Case report

**Figure 1:**
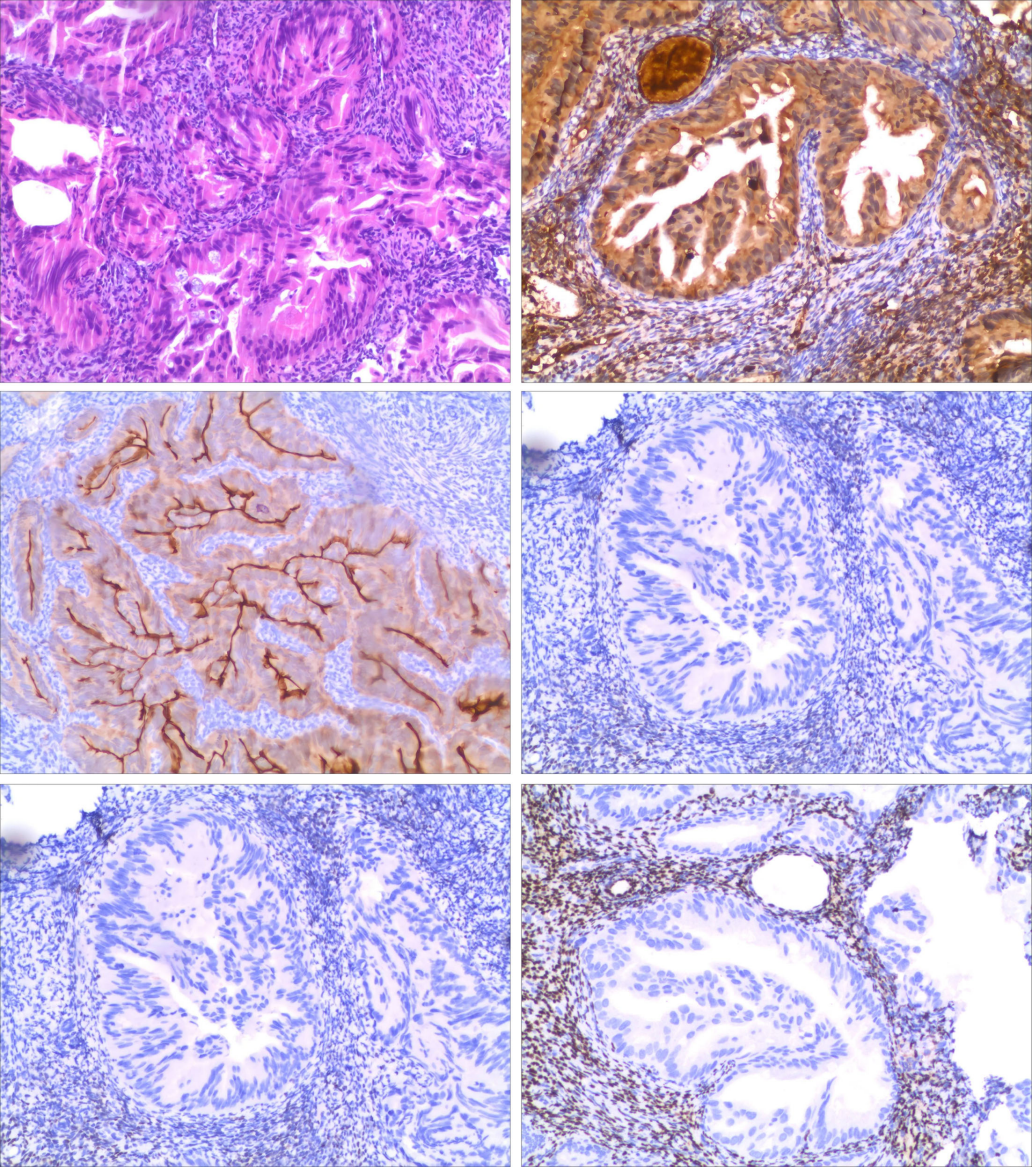
Pathology after diagnostic curettage. Images ① and ② are endometrial biopsy specimens, stained with hematoxylin–eosin (H+E), ×4 and ×10 magnification (respectively for “①” and “②”), showing invasive mucus adenocarcinoma. The patient’s previous pancreatic cancer is morphologically similar to that shown here. Images ③, ④, ⑤, and ⑥ are immunohistochemical stains for antiproteinase (AAT+), human chorionic gonadotropin 7 (Villin+), estrogen receptor (ER−), and progesterone receptor (PR−) on endometrial excision specimens, ×10.

**Figure 2:**
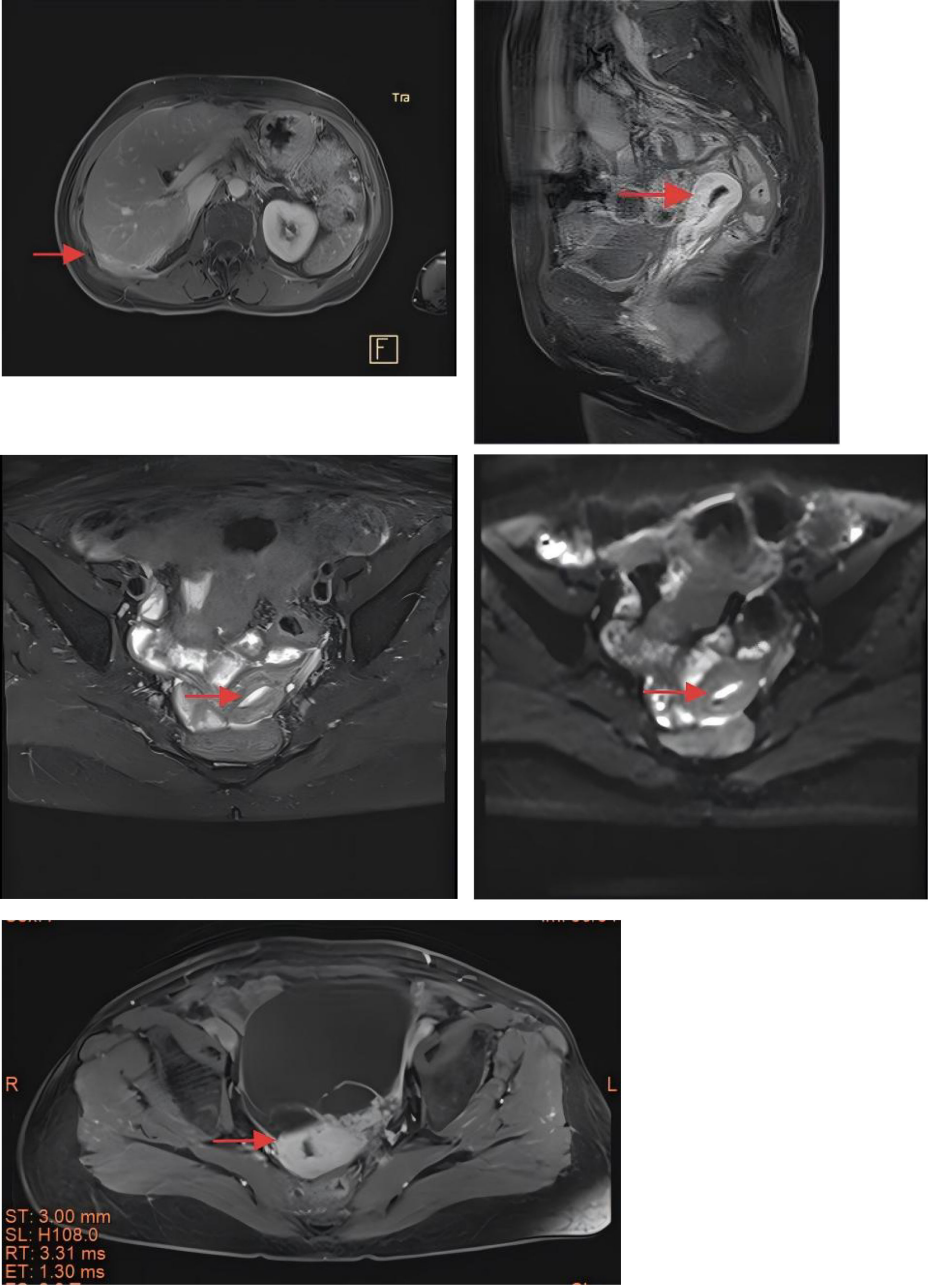
Metastatic tumors of the liver and uterus. ① is a liver metastatic tumor; ②, ③, and ④ are endometrial metastatic tumors, with the endometrium thickened in a ring shape. DWI shows diffusion limitation and significant delayed enhancement on the enhanced scan; ⑤ is a metastatic giant tumor of the uterus and ovary.

**Figure 3:**
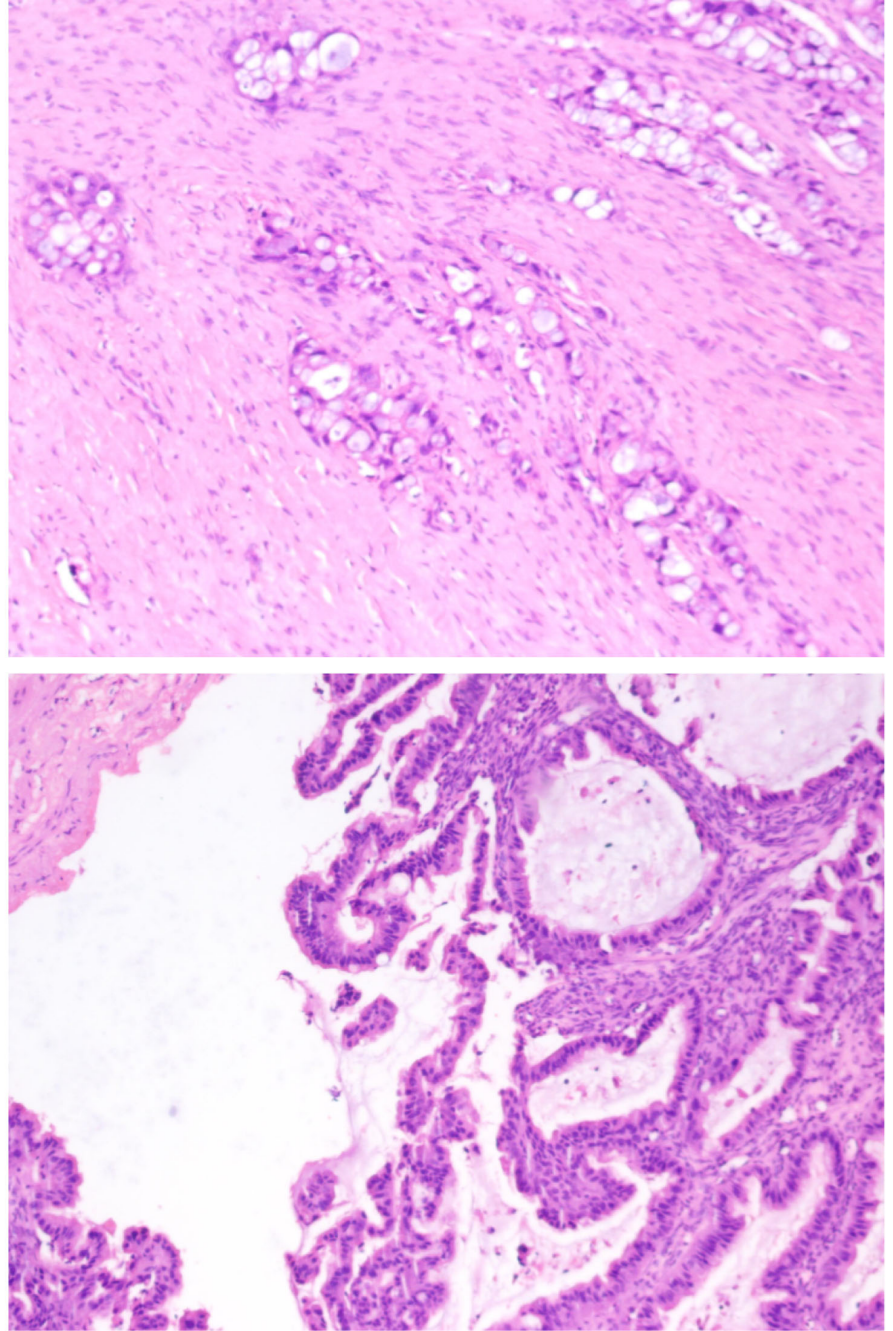
Pathology after total hysterectomy with bilateral salpingo-oophorectomy.

A 66-year-old woman with a family history of death from pancreatic cancer (father) visited our hospital with abdominal pain that had persisted for half a year. She underwent an enhanced abdominal computed tomography (CT) scan on 26 August 2019, which revealed an irregular low-density shadow in the head of the pancreas, measuring approximately 1.8 cm × 2.4 cm, with no clear enhancement on the scan. The lesion was connected to the main pancreatic duct, which was notably dilated. The dilation of the main pancreatic duct was most pronounced at the head and neck of the pancreas, with the widest part measuring 2.3 cm. The pancreatic parenchyma was hypodense with blurred edges. The diagnosis was an irregular cystic lesion in the head of the pancreas with dilation of the main pancreatic duct and was considered to be an IPMN; malignancy was excluded based on clinical findings. The upper abdominal magnetic resonance imaging (MRI) showed that the outline of the pancreas was clear but irregular; the main pancreatic duct was significantly widened, particularly in the head, neck, and body, with the widest diameter measuring approximately 1.8 cm. A tortuous tubular shadow was visible, communicating with the main pancreatic duct at the hook portion, and a 1.7-cm round cystic shadow was observed in the pancreatic neck, which appeared to communicate with the main pancreatic duct. The diagnosis was main pancreatic duct dilation, a tortuous tubular structure in the pancreatic neck, and a cystic shadow in the pancreatic neck, indicating a possible pancreatic IPMN. MRCP: The intra- and extrahepatic bile ducts, gallbladder, and pancreatic duct are shown. The intrahepatic bile ducts were dilated, and no abnormal signals were seen in the lumen. The common bile duct was slightly widened, with uneven thickness and a diameter of approximately 0.9 cm at the widest point. The main pancreatic duct was uneven in thickness and diameter, with obvious dilation in the local area; the maximum diameter of the pancreatic duct in the head and neck of the pancreas was approximately 1.9 cm. Cystic mixed signals could be seen in the neck of the pancreas with a diameter of approximately 1.4 cm, which seemed to be connected with the main pancreatic duct in the local area. The pancreas was atrophied, with a few cords seen in the peripancreatic area. Diagnostic comments: Cystic abnormal signal in the neck of the pancreas, possible IPMN, marked dilation of the main pancreatic duct, pancreatic atrophy, uneven thickness of the common bile duct, and slight dilation of the intrahepatic bile duct. Laboratory tests revealed the following: CA19-9: 6.70 U/mL (0.00–35.00 U/mL), TBil: 8.41 µmol/L, DBil: 1.44 µmol/L, and IBil: 6.97 µmol/L. The patient underwent “total pancreatectomy” 4 weeks after the imaging diagnosis. Postoperative pathology revealed high-grade dysplasia of the IPMN with invasive cancer (malignant tumor focus under the microscope with a maximum diameter of approximately 0.7 cm) in the proximal duodenum and gallbladder of the pancreas. The cancer invaded the pancreatic tissue but did not invade the duodenal or gastric walls. No vascular invasion was detected, and no cancer was detected in the gastric margin, duodenal margin, or pancreatic margin. A pancreatic body tail IPMN showed high-grade dysplasia of the epithelium. Four lymph nodes in the duodenal lymph node group and Group 8 lymph nodes were not found to have cancer metastasis. Surgical staging: T1b N0 M0, IA stage. Six cycles of “gemcitabine + S-1” regimen chemotherapy were administered after surgery, followed by regular follow-up. In May 2021, the patient experienced waist and right costal pain. An MRI of the liver revealed a focal abnormal signal under the dorsal membrane of segment VIII of the right lobe of the liver. Considering liver metastasis, two cycles of nab-paclitaxel combined with nedaplatin chemotherapy were initiated, but the therapeutic efficacy was not evaluated, and the patient discontinued antitumor therapy on his own because of nausea and vomiting. In April 2023, the patient presented to a gynecologist with vaginal bleeding for no apparent reason. A vaginal gynecological ultrasound revealed that the muscular layer of the uterine wall was echogenic and homogeneous, the endometrium was centered, and the thickness of the single layer was approximately 0.1 cm. A liquid dark area measuring 1.7 cm × 0.8 cm × 1.1 cm could be seen in the uterine cavity. There was no obvious abnormality in the bilateral adnexal region. Color Doppler flow imaging (CDFI) did not show any abnormal blood flow signals. Four weeks later, a hysteroscopy with diagnostic dilation and curettage (D&C) was performed under combined spinal–epidural anesthesia. During hysteroscopy, the uterus was anterior and normal in size, with no abnormalities in the bilateral adnexa. The hysteroscope entered slowly, and the cervical opening was partially adherent. After dilation, the cervical canal appeared normal in shape. A small amount of fluid was observed in the uterine cavity, the endothelium of the posterior wall was disorganized, and inflammatory congestion was noted at the oozing points. A small amount of endothelial tissue was scraped from its root with a ring-shaped electrosurgical instrument for biopsy, and the excised material was sent for pathological examination. Pathology (uterine cavity): Endometrial atypical cell clusters, consistent with endometrial metastatic pancreatic cancer based on morphology and immunohistochemical results. Immunohistochemical results: ER (−), PR (−), PAX-2 (−), Ki-67 (+, approximately 20%), P16 (+), vimentin (−), AAT (+), S-100 (−), Mucin-5AC (−), VIM (−), CA125 (+), CA199 (+), CK19 (+), fascin (−), SMAD4 (+), DPC-4 (+), S100p (−), and Villin (+) (**Figure 1**). Enhanced MRI revealed that the subcapsular lesion in segment VIII of the liver had increased in size compared with before, with high signal intensity on T2W1 and low signal intensity on T1W1. There was slight ring-like enhancement during the enhanced scan, suggesting a high possibility of metastasis. Enlarged lymph nodes in the retroperitoneum were noted; the endometrium showed concentric thickening, with restricted diffusion on DWI and significant enhancement during the delayed enhanced scan, indicating possible metastasis (**Figure 2**). On 29 May 2023, PET–CT indicated poor imaging of liver and endometrial metastases, revealing multiple soft tissue nodules and masses in the peritoneal and mesenteric areas with increased metabolism, suggesting metastasis. Tumor biomarkers: CA 19-9–440 U/mL. The MDT made a diagnosis of pancreatic cancer with stage IV liver, endometrial, and peritoneal metastases post surgery, with no surgical option. The germline mutation results revealed the following: KRAS exon 2 G12R mutation, BRCA1/BRCA2 no mutation, and microsatellite status MSS. Systemic treatment is recommended. The chemotherapy regimen included 200 mg of irinotecan on Day 1 + 130 mg of oxaliplatin on Day 2 + 3,750 mg of 5-FU continuously infused for 46 h, which was repeated every 14 days for six cycles. Imaging evaluation revealed stable disease (SD), while the patient’s serum CA 19–9 concentration remained elevated. During this period, the patient experienced Common Terminology Criteria for Adverse Events grade 3 gastrointestinal reactions and fatigue as side effects of chemotherapy. The regimen was changed to maintenance chemotherapy with 110 mg of liposome irinotecan for four cycles. Pelvic MRI indicates the disappearance of endometrial enhancement and stable liver lesions. The patient did not undergo regular follow-up for family reasons. The patient subsequently presented with lower abdominal distension and vaginal bleeding. Enhanced MRI on 18 June 2024 revealed a cystic lesion in the left adnexal area that had significantly increased since 29 February 2024, with a thickened and irregularly shaped endometrium. Considering the patient’s history, metastasis was suspected. After MDT consultation, debulking surgery was recommended. Three weeks later, total hysterectomy and bilateral adnexectomy were combined with peritoneal hyperthermic perfusion chemotherapy. Intraoperative findings include (left adnexa) a grayish-white and grayish-red cystic mass, measuring 9 × 7 × 6.5 cm, with a cystic wall thickness of 0.2–0.6 cm in section; the inner wall was rough, granular, and hard. A total resection specimen of the uterus and right adnexa was dissected along the anterior wall in the shape of a Y, with the uterus measuring 8.5× 5 × 3.5 cm. The uterine cavity was 6.5 cm deep, the wall was 1–1.5 cm thick, and the inner lining was 0.2–0.5 cm thick, with local bulging; the cervix was smooth. Postoperative pathology revealed adenocarcinoma in the left ovary, left fallopian tube, endometrium, abdominal wall tumor, and interintestinal tumor, which was consistent with metastatic pancreatic cancer based on morphology, history, and immunohistochemistry (**Figure 3**). The intrahepatic lesions were stabilized on MRI at 1 month after surgery; no new lesions were seen in the abdominopelvic cavity. The patient is now being treated with anlotinib maintenance therapy, and the survival period is now more than 5 years.

## Discussion

IPMN is a rare exocrine pancreatic tumor that was first reported by Takagi et al. in Japan in 1982 and accounts for approximately 1% to 3% of pancreatic tumors (1). Based on the type of pancreatic duct involvement, IPMNs can be classified into main duct intraductal papillary mucinous neoplasm (MD-IPMN), branch duct intraductal papillary mucinous neoplasm (BD-IPMN), and mixed-type intraductal papillary mucinous neoplasm (MT-IPMN). Radiologically, MD-IPMNs often present with significant dilation of the pancreatic duct and are typically associated with an increased risk of malignant transformation, leading to a poor prognosis. In contrast, BD-IPMNs are more commonly seen as cystic lesions in the head of the pancreas and are associated with a lower risk of malignancy, whereas MT-IPMNs fall between the two (2, 3). IPMN progresses slowly, from low- to high-grade dysplasia to IPMN-associated invasive carcinoma. Primary duct IPMN (MD-IPMN) with invasive carcinoma is more common, with postoperative pathology reports indicating that approximately 62.1% to 71.0% of cases exhibit high-grade dysplasia or invasive carcinoma (4, 5). Therefore, surgical resection is a key treatment strategy for patients meeting high-risk criteria, such as those outlined in the Fukuoka Consensus. However, even after surgical treatment, invasive IPMNs are associated with a recurrence risk of approximately 45% (6). Nevertheless, its prognosis remains better than that of classical pancreatic ductal adenocarcinoma (7), with a 5-year survival rate ranging between 39% and 90% (8), and the intestinal-type colloid subtype has a more favorable outcome.

Endometrial metastases originating from extragenital primary tumors are exceedingly rare. The uterus is a relatively small organ with minimal distal blood flow and contains abundant fibrous and smooth muscle tissue. Additionally, the periodic shedding of the endometrium may hinder the implantation of circulating tumor cells (9). This process can impede tumor dissemination. It has been reported that uterine metastases primarily involve the myometrium, whereas endometrial metastasis is even rarer; often, by the time they are detected, they are already accompanied by extensive metastases in other sites, such as the lymph nodes, lungs, liver, and bone. Moreover, most previously reported cases were identified in autopsy specimens rather than surgical pathology specimens, which may suggest delayed or inaccurate diagnosis and a lack of targeted treatment (10–12). Primary tumors that can lead to such metastases include breast cancer, colorectal cancer, lung cancer, and melanoma, with cases of pancreatic cancer also reported in pathological diagnoses (12–16). Pancreatic cancer is highly malignant and prone to local infiltration and distant metastasis. Stemmermann (17) proposed that the mechanism of endometrial metastasis is largely attributable to local lymphatic spread from ovarian involvement and hematogenous metastasis. Pancreatic cancer can metastasize early to high-level lymph nodes, such as the para-aortic and common iliac nodes. If these lymph nodes are obstructed by tumor cells, cancer cells may disseminate retrogradely via lymphatic vessels or through collateral circulation into the pelvic lymph nodes, subsequently invading the uterine body and endometrium via the utero-pelvic ligament lymphatic network. Conversely, once pancreatic cancer cells invade the portal vein or systemic circulation, they can also disseminate to pelvic organs via the bloodstream. Huo et al. (18) reported the presence of neoplastic emboli within blood vessels in surgical specimens. In this case, the patient’s ovaries were not involved at the initial diagnosis of endometrial metastasis, and simultaneous liver metastasis was present, suggesting that hematogenous spread likely played a major role. Subsequent ovarian involvement may be related to local invasion. The patient’s initial symptom was vaginal bleeding, which is consistent with reports in the literature (19, 20). The diagnosis of endometrial metastasis relies primarily on the patient’s history of the primary tumor, histopathological tissue biopsy showing similar tumor cells to the primary tumor, and immunohistochemical phenotype. When metastasis occurs in the endometrium, it typically appears within the stroma adjacent to normal endometrial glands. In this case, the tumor cells were observed in the endometrial curettage tissue to be columnar, pseudostratified, and irregularly growing, with a mucus visible within the cells. Genetic testing revealed a Kras mutation, which was consistent with the morphology and genetic phenotype of pancreatic cancer, differing from the morphology of primary endometrial adenocarcinoma. Immunohistochemistry revealed estrogen receptor (ER) negativity, progesterone receptor (PR) negativity, Villin positivity, and AAT positivity, which is consistent with the immunohistochemical manifestations of pancreatic cancer and identification of it as a primary endometrial cancer.

In terms of treatment, IPMN-associated pancreatic cancer is similar to classic pancreatic cancer. As mentioned earlier, surgery is the primary approach for early-stage patients, whereas systemic therapy serves as the mainstay for advanced cases. For patients with metastatic endometrial cancer, the presence of metastasis often indicates widespread dissemination of the disease and is associated with poor prognosis (11, 17). Previous reports have suggested that if no other extrapelvic metastatic lesions are detected, surgery may interrupt the metastatic cascade and help prolong disease-free survival (13, 18, 21). For patients with metastatic disease, systemic therapy remains the mainstay of treatment, with chemotherapy, radiotherapy, and targeted therapy all reported to prolong survival (15, 18, 22, 23). The patient’s treatment exemplifies the benefits of personalized treatment and MDT. The primary tumor was pancreatic cancer, with liver metastasis existing before the discovery of endometrial metastasis and concurrent peritoneal metastasis, which precluded surgical resection. The traditional treatment regimen of irinotecan combined with oxaliplatin, fluorouracil, and calcium folinate was poorly tolerated by the patient. Compared with traditional irinotecan, liposomal irinotecan has stronger stability, a longer action time in the blood, and a high permeability and long residence (EPR) effect, leading to low toxicity and high efficacy. The patient achieved a rapid decrease in levels of the tumor marker CA199 and the disappearance of endometrial lesions through single-agent maintenance chemotherapy, with a PFS close to 1 year. The patient subsequently developed a large tumor in the ovarian region of the abdomen, which compressed the pelvis, along with the progression of endometrial lesions and extensive peritoneal metastasis. Referring to the peritoneal cancer index (PCI) score for peritoneal metastatic carcinoma of the digestive tract, the upper right abdominal tumor was given a score of 2 points, the pelvic tumor was given a score of 3 points, the lower right abdominal tumor was given a score of 3 points, and the interintestinal mass was given a score of 2 points, totaling 10 points (<20 points), which met the criteria for cytoreductive surgery (CRS). Additionally, successful cases of CRS in patients with uterine metastasis are available for reference (24). However, owing to the high degree of malignancy and poor prognosis of pancreatic cancer, CRS is not typically recommended under normal circumstances. Moreover, this patient also had liver metastasis. However, considering expert opinions, the patient’s IPMN with infiltrating cancer grew slowly, with insignificant changes in liver lesions over 3 years. Patients with gelatinous intestinal-type pancreatic cancer have relatively longer survival times but are less sensitive to chemotherapy. Given the patient’s good condition and suitability for surgery, comprehensive CRS was performed. Several previous trials have confirmed that combined heat infusion chemotherapy after CRS improves the prognosis of patients with malignant tumors. For instance, in 2018, the *New England Journal of Medicine* reported that Dutch researchers van Driel et al. (25) reported that simple CRS could achieve a survival period of 34 months in patients with ovarian cancer, whereas when combined with cisplatin, the survival period could be extended to 46 months. In a patient with gastric cancer with malignant ascites, the role of HIPEC is more pronounced, with an effective rate of up to 50% (26). Therefore, this patient was given combined intraperitoneal heat-perfusion cisplatin chemotherapy, and at 6 months postoperatively, he had resumed maintenance systemic therapy and had a good quality of life. His overall survival has exceeded 5 years with the combination of surgery, chemotherapy, and other treatment modalities.

Reporting this case highlights the possibility of secondary endometrial metastasis in a patient with IPMN with pancreatic infiltrating cancer for gynecologists and oncologists. Comprehensive imaging to assess disease stage and biopsy of metastatic lesions to obtain a pathologic diagnosis are essential for effective tumor management. Combined tumor reduction based on systemic therapy may improve patient survival.

## Data Availability

The datasets presented in this study are not publicly available due to restrictions imposed by the ethical approval and institutional policies governing this research. Requests to access the datasets should be directed to the corresponding author, YG, at 15006525379@163.com.
