# Supplementary figures and images for: Pancreatic intraductal papillary mucinous neoplasm with invasive carcinoma and uterine metastasis: a case report
Source: Front Oncol. 2026 Feb 24;15:1562588. doi: 10.3389/fonc.2025.1562588 (PMC12971412; doi:10.3389/fonc.2025.1562588)

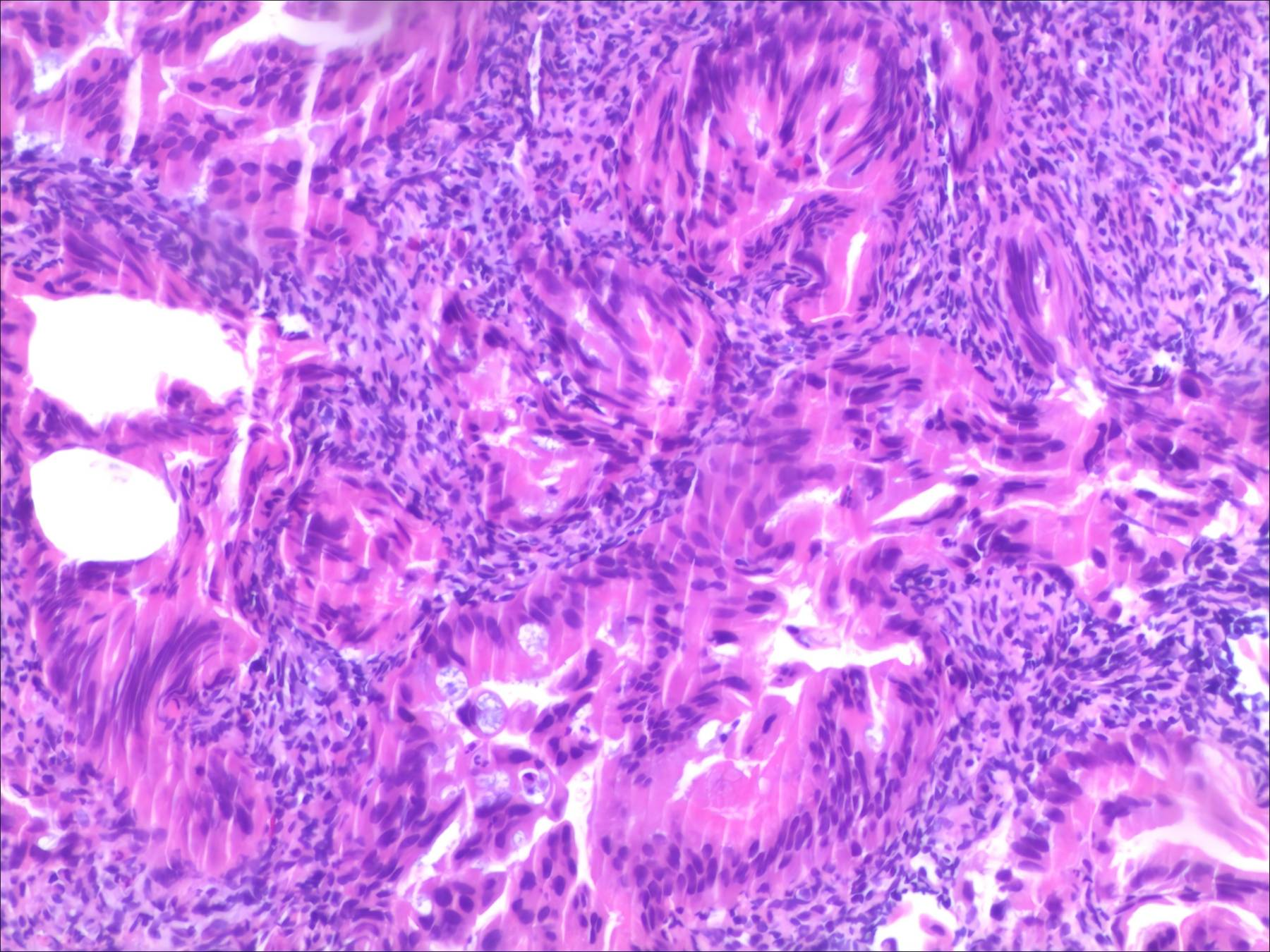

Supplement: Supplementary file 1 [file Image1.tiff]

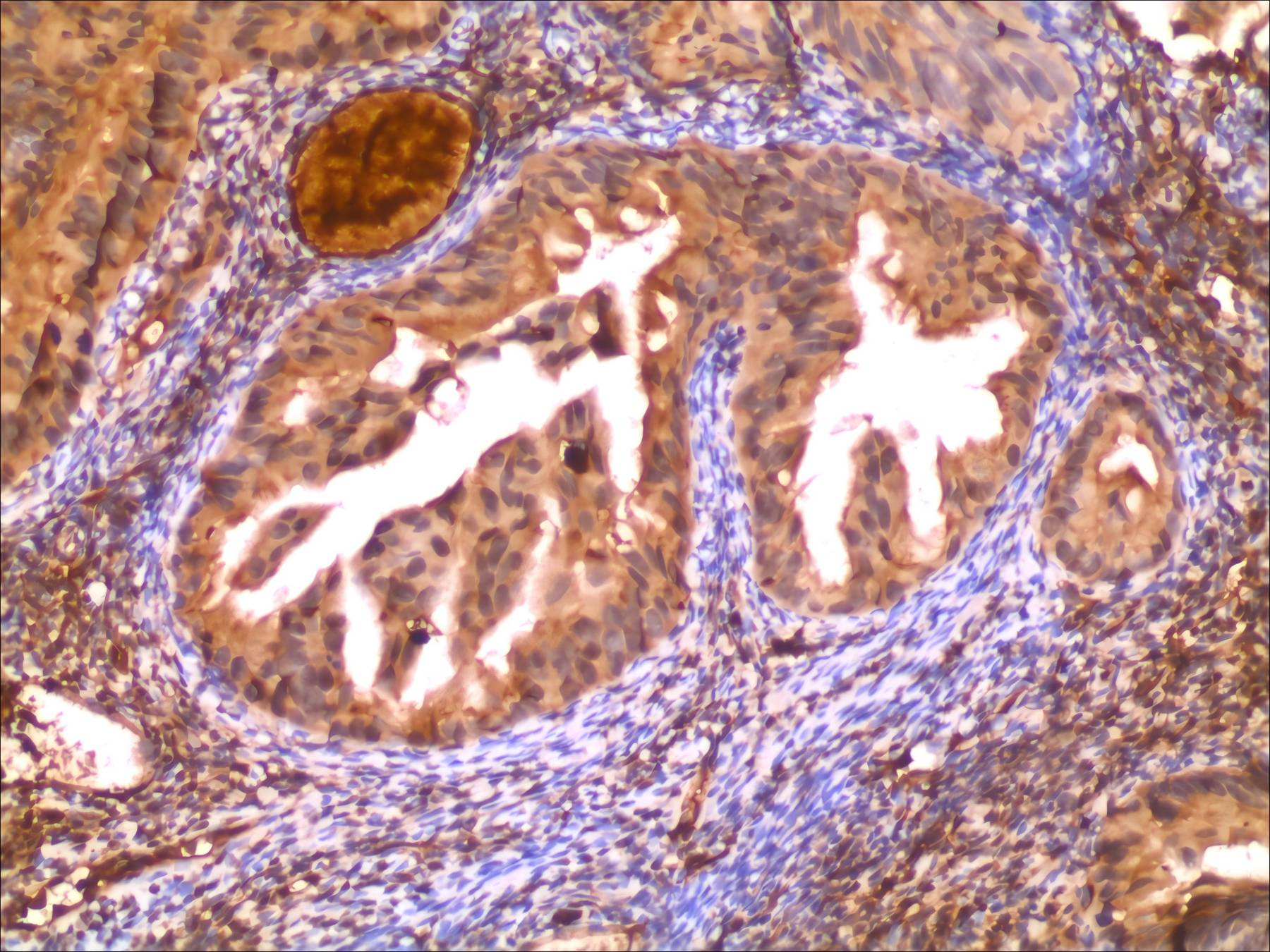

Supplement: Supplementary file 2 [file Image2.tiff]

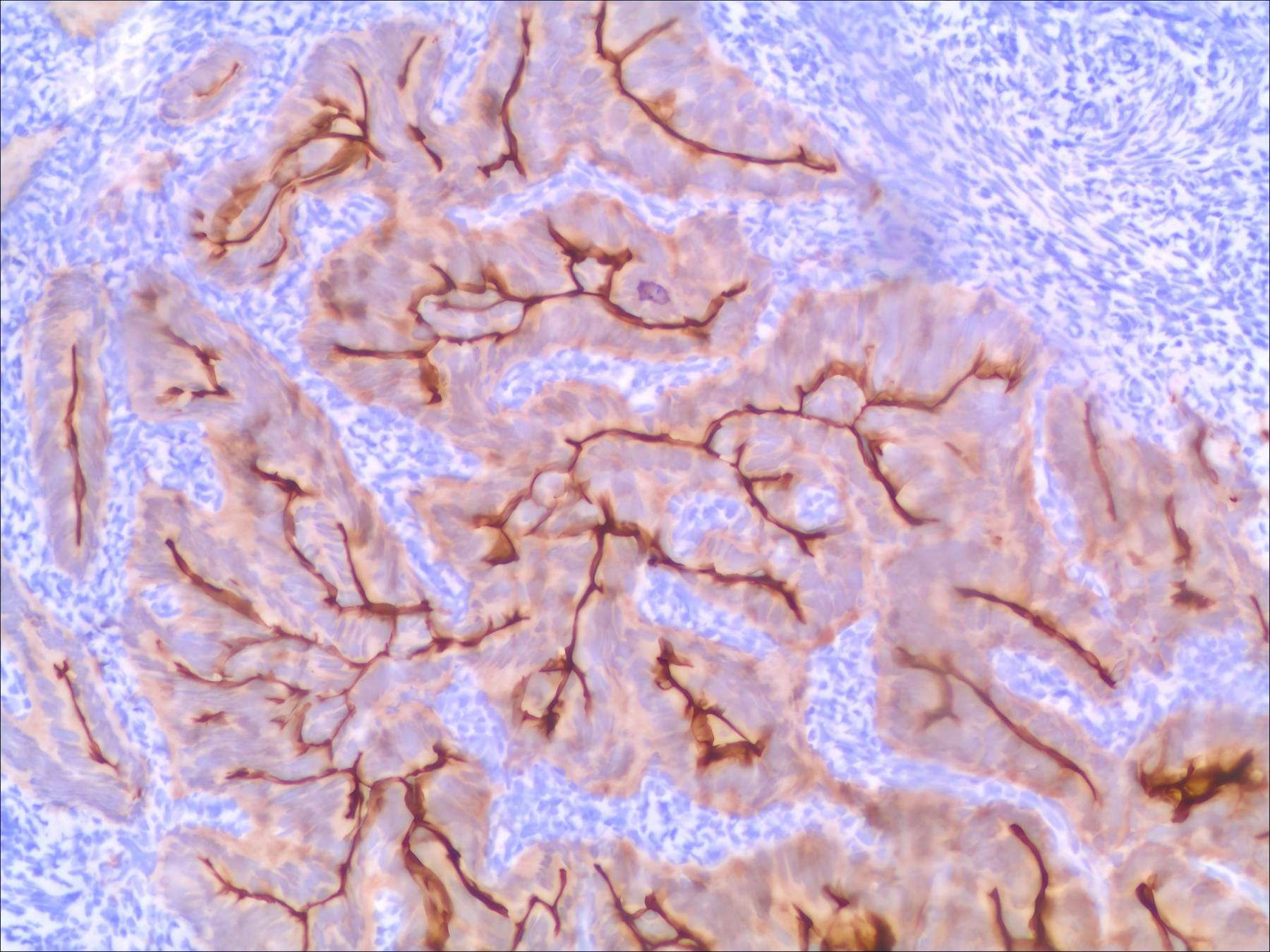

Supplement: Supplementary file 3 [file Image3.tiff]

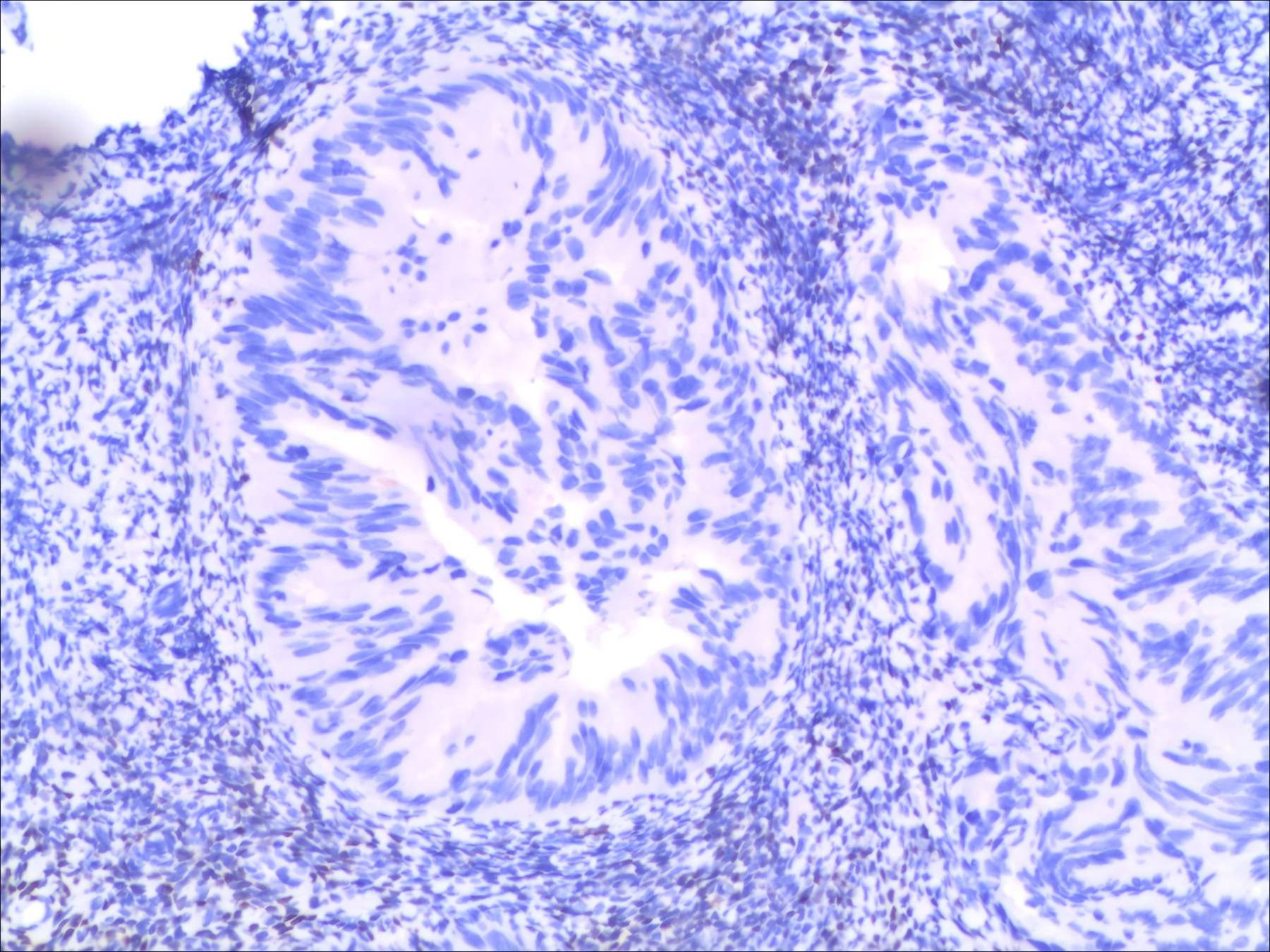

Supplement: Supplementary file 4 [file Image4.tiff]

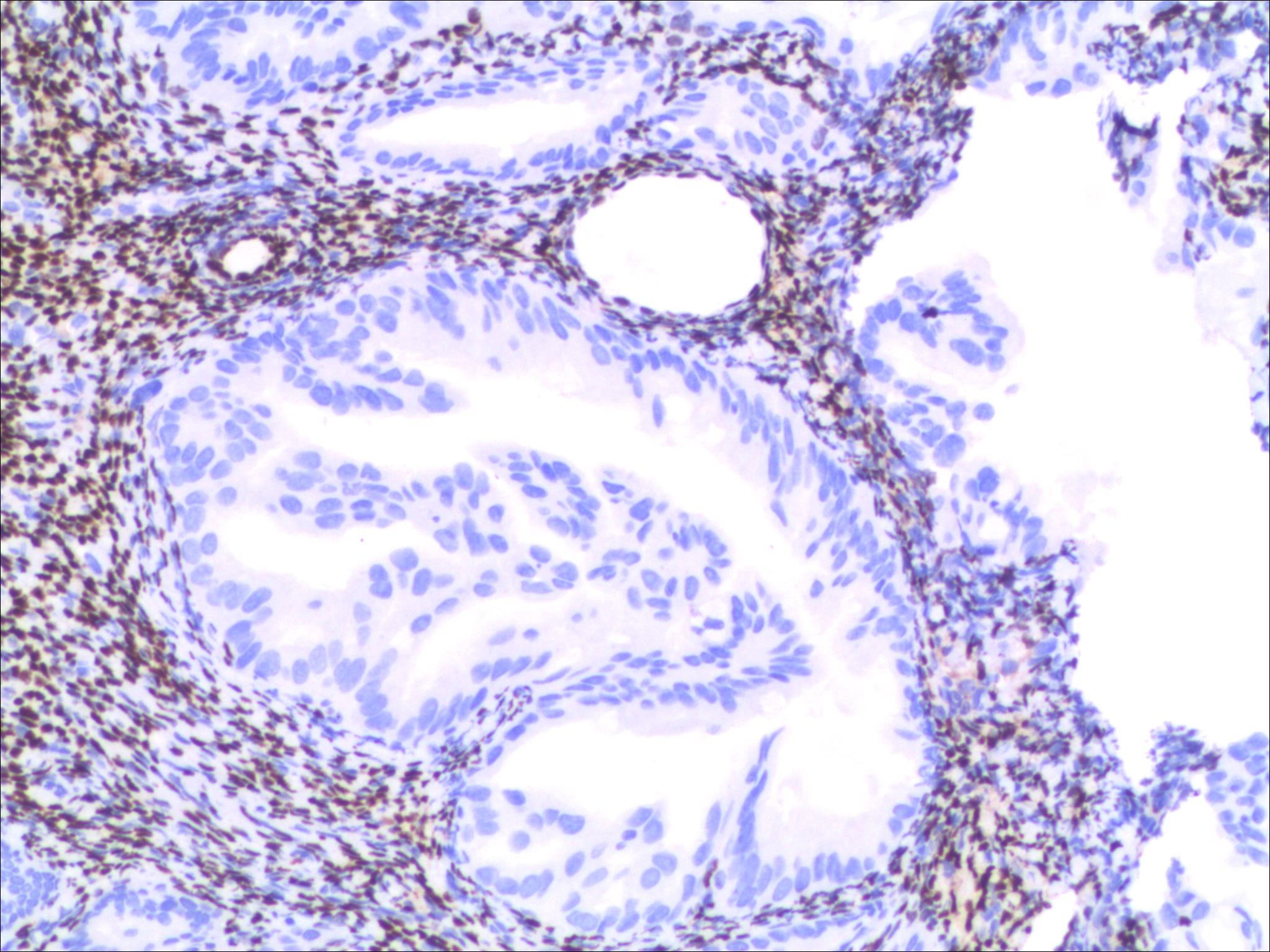

Supplement: Supplementary file 6 [file Image6.tiff]

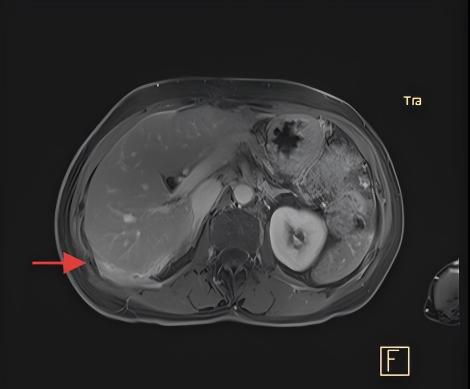

Supplement: Supplementary file 7 [file Image7.jpeg]

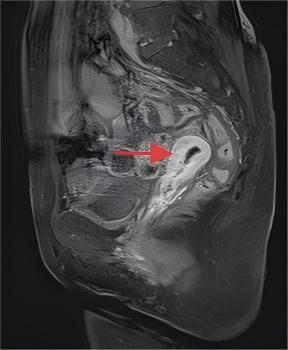

Supplement: Supplementary file 8 [file Image8.tiff]

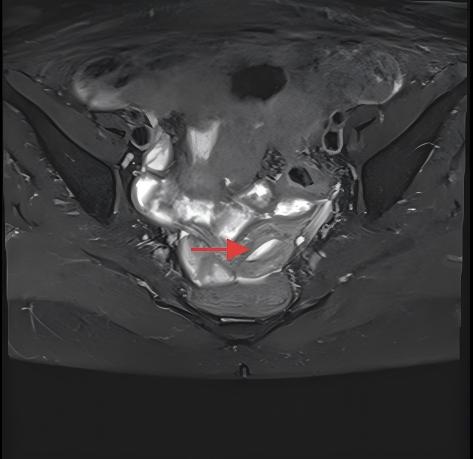

Supplement: Supplementary file 9 [file Image9.tiff]

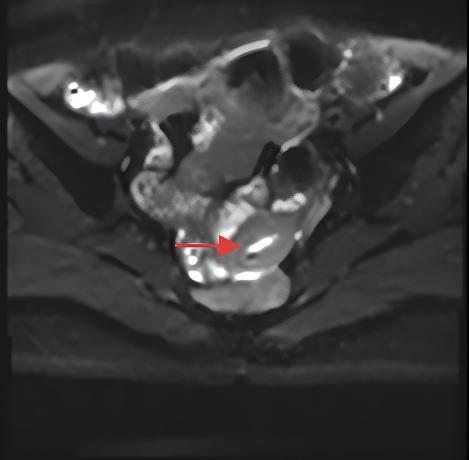

Supplement: Supplementary file 10 [file Image10.tiff]

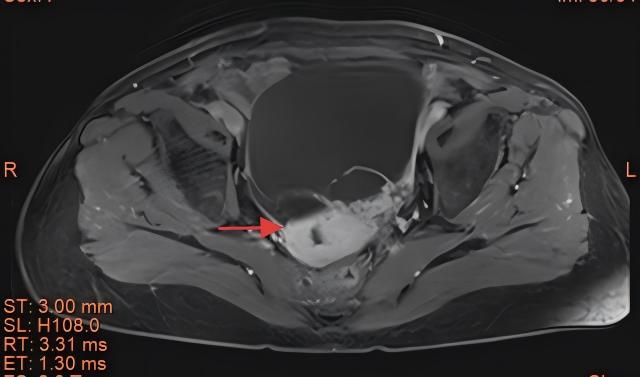

Supplement: Supplementary file 11 [file Image11.tiff]

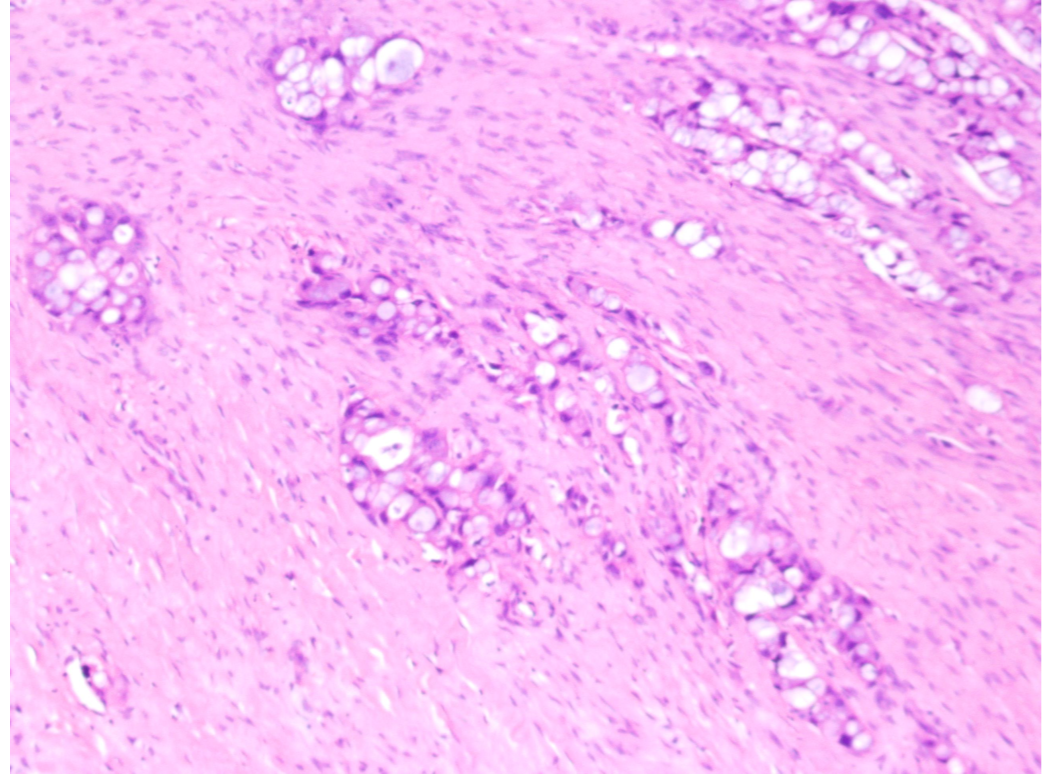

Supplement: Supplementary file 12 [file Image12.tiff]

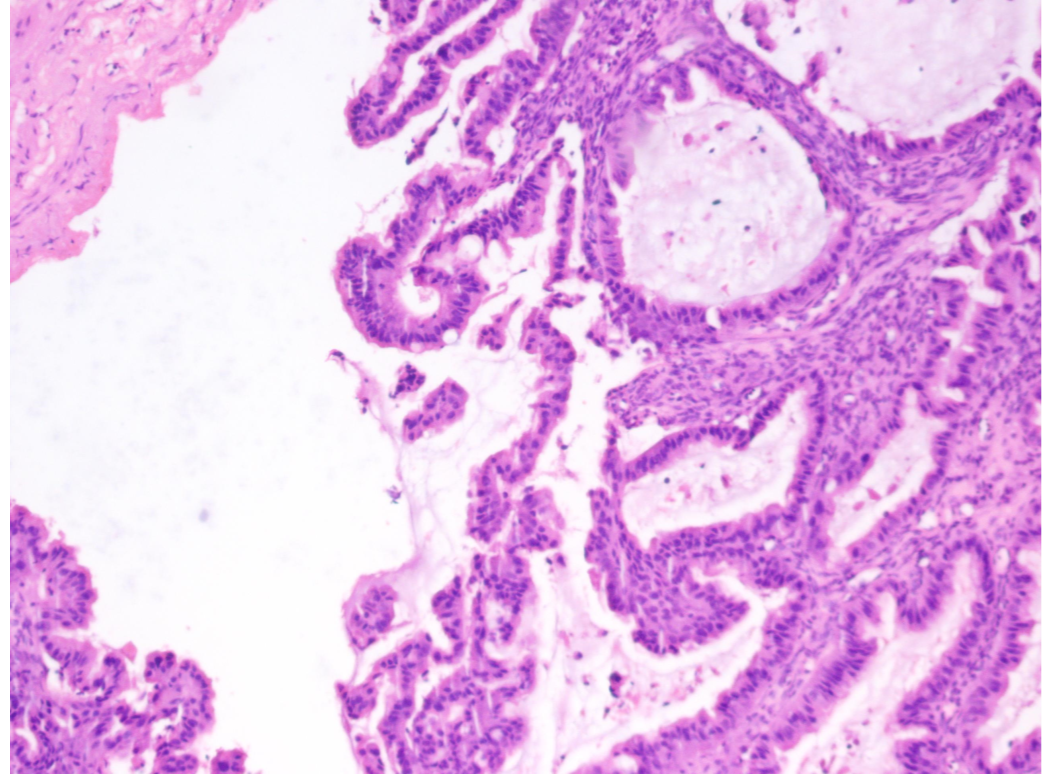

Supplement: Supplementary file 13 [file Image13.tiff]
